# Quantum dot: a next-generation tool for cancer diagnosis at an early stage

**DOI:** 10.1186/s43046-025-00329-4

**Published:** 2025-11-09

**Authors:** Adhi Kesava Naidu Neelam

**Affiliations:** 1 Shri Vishnu College of Pharmacy, Bhimavaram, India; 2 Andhra University, Visakhapatnam, India

**Keywords:** Quantum dots (QDs), Graphene QDs, Nanomaterials, Nano-delivery vehicles, Chemotherapy, Novel treatment

## Abstract

**Background:**

The convergence of biology and nanomaterials has propelled technological progress in biomedical sciences, offering transformative applications in diagnostics and therapy. Among these advancements, quantum dots (QDs) semiconductor nanocrystals activated by light have emerged as versatile tools due to their unique optical and electronic properties. Graphene quantum dots (GQDs), a subset of QDs, are nanoscale fragments of graphene that exhibit exceptional features, making them highly suitable for innovative biomedical applications. These include cancer detection, drug delivery, and imaging, areas where early diagnosis and effective treatment are crucial.

**Main body:**

The production of synthetic GQDs relies on two primary approaches: top-down methods, where larger carbon structures are broken into smaller fragments, and bottom-up methods, which involve assembling GQDs from smaller molecular units. Both methods offer advantages depending on the desired properties and applications of the GQDs. GQDs possess several beneficial characteristics, including high photostability, excellent biocompatibility, and tunable fluorescence, which make them particularly valuable for biomedical purposes. In cancer therapy, GQDs serve as efficient nano-delivery vehicles for drugs, offering enhanced targeting and reduced side effects compared to traditional chemotherapy. Furthermore, their fluorescence properties enable precise imaging and early detection of cancerous cells, providing a dual functionality in diagnosis and therapy. Current research highlights advancements in QD synthesis techniques, enhancing their scalability and application potential. These innovations underscore the role of GQDs in bridging the gap between experimental research and clinical applications.

**Conclusion:**

Quantum dots, particularly graphene quantum dots, represent a breakthrough in the field of nanomedicine. Their synthesis, functional properties, and dual roles in diagnostics and therapeutic delivery underscore their importance in advancing cancer treatment and early detection. With continued research and development, GQDs are poised to revolutionize drug delivery systems and expand the horizons of biomedical science.

## Introduction

Quantum dots, a semiconductor nanocrystal, glow in response to light. Among their outstanding optical qualities are their tremendous brightness, resilience to photobleaching, and changeable wavelength. Novel innovation in quantum dot modification of surfaces opens the possibility of their application in cancer imaging and treatment. QDs are helpful fluorescent bio-probes for studies in biology and medicine. Due to their characteristics, they can be used in vivo as well as in vitro for biological and cellular visualization of cells, which has resulted in breakthroughs in cancer detection and treatment and image-guided chemotherapeutic drug delivery.

Quantum dots have demonstrated hitherto unheard-of potential for enhancing drug loading, targeting, and effectiveness in cancer therapies. Cutting-edge platforms for quantum technology, like carbon quantum dots (CQDs), graphene quantum dots (GQDs), MnCdTeSe/CdS, Gd-lipid in the coating, and CdSe/ZnS/silica, have been used in biosensing, in vitro diagnosis, cancer treatment, bioimaging, and drug delivery. QDs are frequently employed for the essential purpose of using visual analysis to diagnose cancer and deliver biologically active materials into living things with better antitumor efficacy and safety profiles than free drugs. Several issues, including toxicity, need to be overcome before QD technology may be effectively used in clinical medicine and testing for targeting, imaging, and delivering drugs.

Though their names are similar, semiconductor quantum dots (QDs) and graphene quantum dots (GQDs) are quite different regarding their structures and properties. GQDs, or nanoscale graphene particles, consist solely of carbon and typically contain many edges’ functional groups and sp-2 hybridized areas. GQDs are promising for biomedical applications because of their unique properties such as excellent biocompatibility, photostability, low reported toxicity, and fluorescence which can be controlled by quantum confinement and edge effects. QDs, which comprise elements such as heavy metals and chalcogenides, include materials like CdSe and PbS. QDs exhibit strong fluorescence which may have a wide variety of applications in imaging and optoelectronic fields due to their size-mediated optical and electrical properties. However, the presence of heavy metals has raised serious concerns regarding the toxicity of QDs. While QDs use inorganic core–shell structural aspects to increase brightness and stability, GQDs use their carbon-based structural and chemical properties to enhance both multifunctionality and safety. Accordingly, GQDs are an interesting, environmentally friendly, and biocompatible alternative to standard QDs that have limitations related to the toxicity of their material and regulatory attributes.

## Tumor

For a long time, tumors have been a big issue throughout the world. The cornerstone of a cancer prevention and control plan is early detection and full treatment of the illness; in contrast, improper diagnosis and irregular or insufficient anti-cancer medication may cause problems, the spread of the disease, and the creation of drug-resistant cancer. Complete case data on cancer patients is essential for a precise cancer diagnosis as well as for addressing issues with drug-resistant cancer’s genesis and spread.

## Quantum dots in cancer treatment

Semiconductor nanocrystals known as quantum dots glow when excited by light. High luminosity, resistance to photobleaching, and adjustable wavelength are only a few of their exceptional optical qualities. Their possible use in cancer imaging is made possible by recent advancements in quantum dot surface modification. Applications for near-infrared quantum dots might help with surgery and biopsy by facilitating sentinel lymph node mapping. Targeting cancer in vivo could be achievable by the conjugation of quantum dot molecules with biological matter such as peptides and antibodies.

## Detection of cancer

**Fig. 1:**
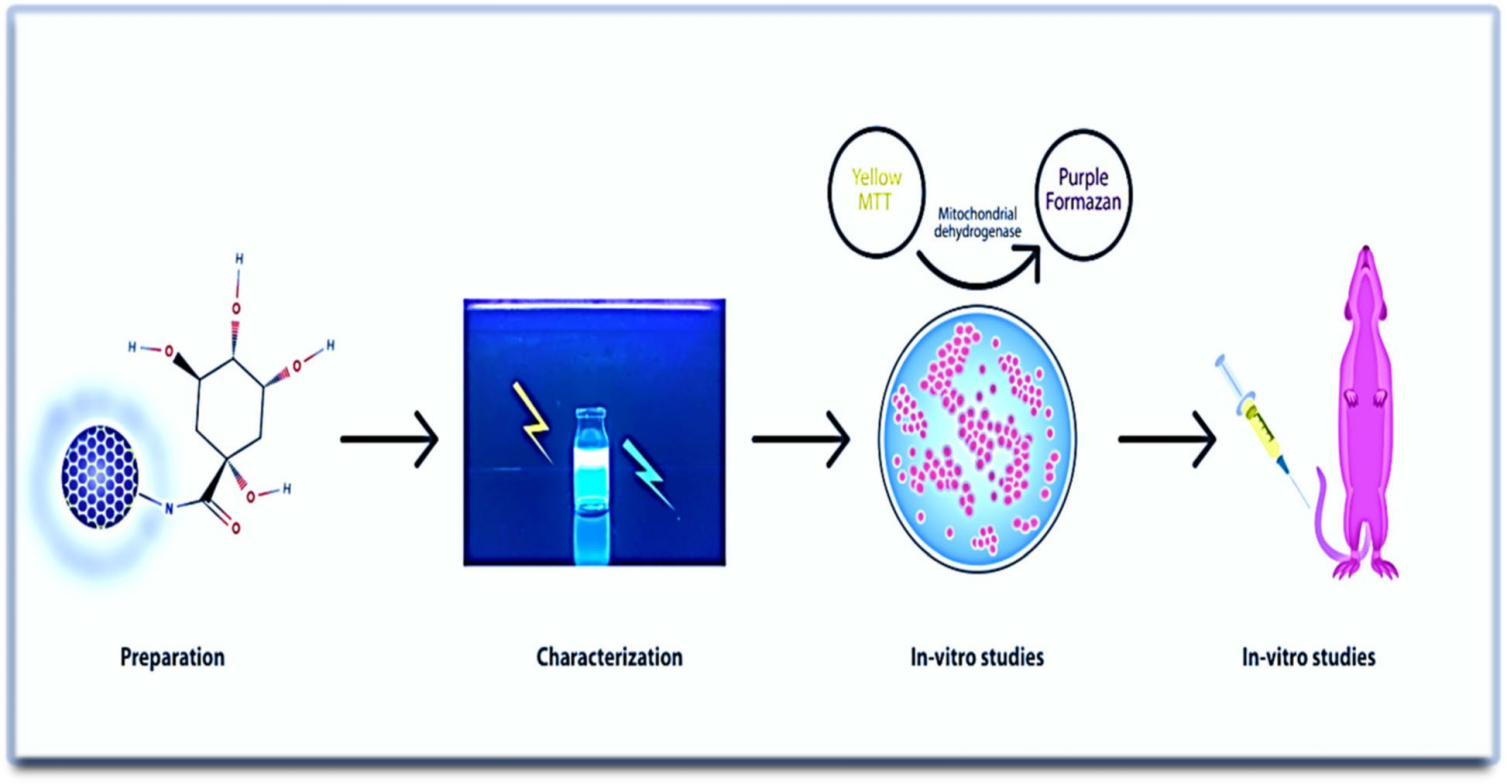
Quantum dot-based cancer detection

When choosing the best cancer treatment, imaging is a crucial therapeutic modality. Modern imaging methods such as computerized tomography, ultrasound, radionuclide imaging, x-ray, and MRI are frequently utilized for monitoring recurrence, assessing the effectiveness of cancer therapy, and screening and staging cancer. Nonetheless, there are two main shortcomings to the imaging methods used today. Firstly, their sensitivity is insufficient to identify the few cancer cells present in the main or metastatic locations. Second, notwithstanding the lack of developed imaging techniques to identify cancer cell-surface markers, they may serve as targets for cancer treatments and aid in the identification and disease staging. Considering these limitations, new extremely sensitive and bio-specific imaging probes must be developed in addition to upgrading the imaging methods now in use. In vivo, cancer imaging can potentially meet these needs with quantum dot detection probes as illustrated in Fig. 1, even if the study is still in its early phases [1–4].

## Graphene quantum dots

Among the most crucial instruments for clinical diagnosis and therapeutic evaluation is fluorescence molecular imaging, which enables the noninvasive, extremely sensitive, and unique monitoring of physiological and pathological phenomena. Many fluorescent agent probes are being investigated constantly. Conventional fluorescent labels known as “organic dyes” have been extensively employed in bioimaging, despite their photobleaching, low solubility, and limited durability.

The high quantum efficiency of their photoluminescence, adjustable emission wavelength, improved photostability, and robust luminescence, semiconductor quantum dots, or QDs, have been considered perfect substitutes. However, because most semiconductor QDs like CdSe, CdTe, and CdS introduce heavy metals, there is growing concern about the possible health and environmental dangers associated with these particles [5, 6]. The interaction among the coating ligands as well as QDs may also have an impact on the photostability of the as-prepared QDs. Furthermore, complex procedures are frequently required to alter the surface of QDs to meet diverse application situations. Graphene quantum dots, or GQDs for short, are a revolutionary 2D nanomaterial made of lateral sizes of graphene nanosheets less than 10 nm. In the last several years, a lot of studies have been conducted on GQDs. Good optical, thermal, and electrical qualities have led to great interest in graphene quantum dots (GQDs) in a variety of fields, including optoelectronics, energy conversion and storage, and bioscience/biotechnology [7]. GQDs have considerable potential for the field of bioimaging because of their fascinating and programmable fluorescence characteristics, outstanding biocompatibility, minimal cytotoxicity, and exceptional physiological stability.

## Medication usage and resistances in cancerous cells

A chemotherapeutic agent’s mechanism of action may make it more successful against some tumors than others, as drugs used to treat cancer must seriously damage the targeted cells. It is imperative to eliminate cancer stem cells to achieve a favorable outcome devoid of malignancy. However, the ability of these stem cells to activate several drug resistance transporters may result in a drug-resistant cell that is constitutively resistant [8]. Perhaps more concerning is the reality that specific cells that constitute tumors have the potential to develop into tumor stem cells [9]. Since a larger percentage of the medication reaches the cancerous cells in these systems, increasing the frequency of apoptosis, nano-delivery vehicles could potentially be more effective compared to alternative therapy options in this field, like traditional chemotherapy. Multiple drug-resistant transporters can be overcome, according to in vitro research, but in vivo, studies have proven to be extremely difficult because of other consequences that might cause premature drug inactivation owing to drug resistance in non-tumor cells. Complications resulting from in vivo studies must thus be resolved. However, despite these persistent problems, well-thought-out medication delivery systems are making headway. It is important to acknowledge that there is still much to learn about many drug resistance mechanisms, and there is constant study and discussion surrounding them [10].

## Graphene QDs

### Properties and biological applications of graphene QDs

One or more (< 10) graphene lattices of Sp2 carbon atoms with a diameter ranging from 3 to 20 nm make up graphene QDs (GQDs), an allotrope of carbon quantum dots (CQDs) [11]. They have relatively lower toxicity and are more ecologically benign than QDs based on cadmium [12]. Like C-dots, GQD PL emission is an excitation-dependent phenomenon, meaning that as the excitation wavelength rises, the PL maximum redshifts. When compared to typical organic or inorganic fluorophores, C-dots and GQDs have two main advantages: (i) non-blinking PL and (ii) strong photostability, making them appropriate for single-molecule tracking and long-time real-time imaging. GQDs’ primary disadvantage over Cd-based QDs is their reduced PLQYs [12, 13]. The synthesis conditions can be made better and hence increase the PLQYs of GQDs. Using a hydrothermal method and ameliorative photo-Fenton reaction, Jiang et al. obtained the highest QY of 24.6% when compared to previous findings on GQDs. Higher QYs were demonstrated to be produced by GQDs’ greater layered structure and crystallinity when compared to naked CQDs. Variations demonstrated that GQDs were suited for biocompatibility and had a greater potential for bioimaging.

In vivo and in vitro cytotoxicity of GQDs was observed by Jiang et al. GQD absorption was confirmed, and it was discovered that they mostly accumulated in the HeLa cells’ cytoplasm. Using CCK-8, LDH, ROS, and annexin V to induce apoptosis as indicated, they observed low levels of ROS in the cells, good cell viability, minimal lactate dehydrogenase (LDH) production, and negligible levels of mortality in the presence and concentration of GQDs (12.5 and 25 mg/mL). Furthermore, GQD distribution in the heart area was demonstrated by in vivo tests conducted on zebrafish embryos and larvae. In these investigations, modest quantities of GQDs (12.5 and 25 mg/mL) were used, and a little variation in heartbeats was noted between the respective groups. These findings demonstrated the limited impact of low concentrations of GQDs on the advancement of the heart area of zebrafish embryos and larvae [14]. Zhang reported on the cellular absorption of unmodified GQDs; however, further GQD functionalization may result in the creation of targeted and detectable genes/DDSs. Furthermore, compared to organic fluorochromes, it has been established that GQDs have better imaging and photochemical stability. Because of the high surface/volume ratio in NPs, the surface chemistry of QDs is essential to their photoluminescence emission process. The existence of OH groups on the top layer of GQDs has been shown by Zhu et al. to cause non-radiative process quenching, which in turn enhances intrinsic state emission and PLQY [15, 16].

### Synthesis approaches

**Fig. 2:**
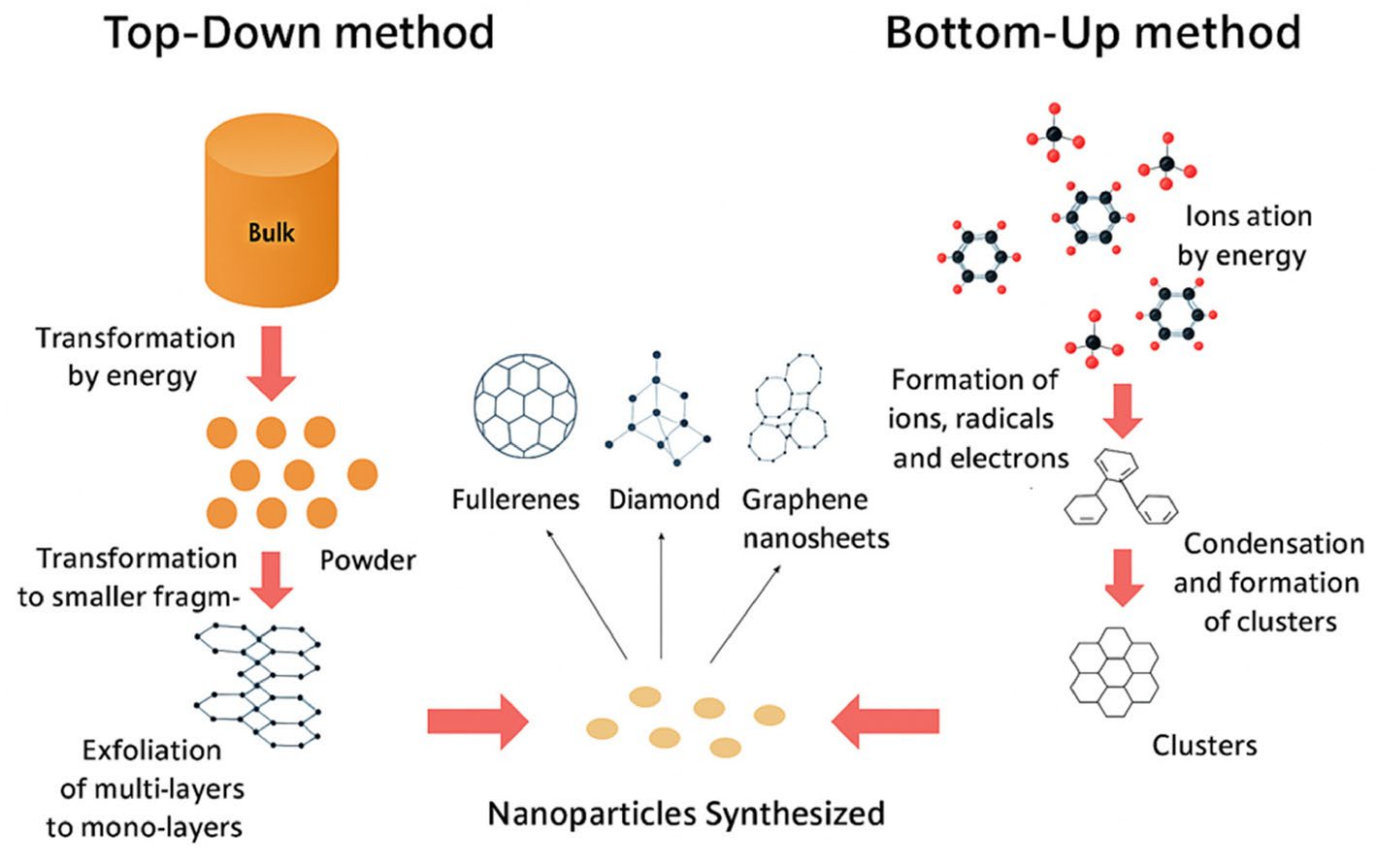
Outline of GQD’s synthesis

Both top-down and bottom-up techniques might be used to synthesize GQDs and CQD’s, which is outlined in Fig. 2. Arc discharge [17], laser ablation [18], and electrochemical techniques [19] are examples of top-down techniques.

Graphite powder, or MWCNTs, which stand for multi-walled carbon nanotubes, is used as a precursor in these methods to manufacture CQDs and GQDs by the application of rough physical or chemical conditions [20]. Additionally, it has been asserted that micrometer-sized pitch-based carbon fibers can be cut and chemically oxidized to create GQDs. Conversely, bottom-up strategies include aqueous and hydrothermal-based processes [21], microwave-aided techniques [22], and thermal pathways [23]. Using microwave pyrolysis, heating, and ultrasonication as external energies, this method uses small molecules like fructose and glucose to create CQDs.

Graphite may be electrochemically exfoliated to produce GQDs, which are then reduced at room temperature using hydrazine. Applying Confocal Fluorescence Microscopy, they demonstrated the effective absorption of the synthesized GQDs by stem cells [12].

The physicochemical characteristics of CQDs and GQDs are determined by the synthesis technique and precursors used, which should be highlighted. The absorbance band morphologies of CQDs made from glucose, for example, vary and are around 250–300 nm in size when produced utilizing hydrothermal, microwave thermal, and ultrasonic techniques [24]. Moreover, various optical characteristics originate from many functional groupings on CQDs [25]. Hence, suitable synthesis techniques should be carried out with correct optimization to acquire the ideal optical characteristics to obtain the required absorbance and PL qualities for authorized uses.

### Quantum dot-based cancer detection

**Fig. 3:**
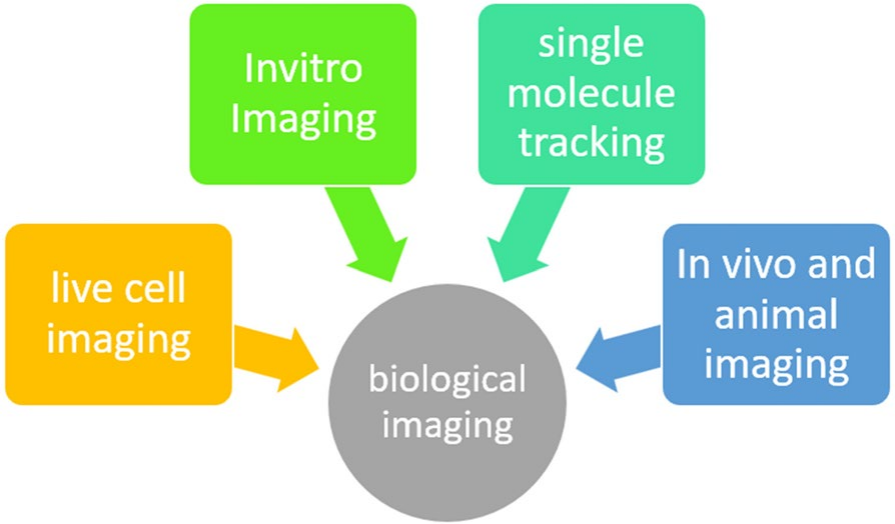
Quantum dot biological uses in monitoring cells and imaging

Research from the past indicates that several researchers can employ QDs in multiplexed imaging and open new applications as bioimaging tools as shown in Fig. 3. Owing to their photostability, they are perfect for examining numerous activities in live cells and for multicolor imaging. For precise and early cancer cell identification, dendrimer/QD nanocrystals (NCs) were used as an ECL signal probe [26]. Because the dendrimer NCs have many functional amine groups, this permitted the assembly of an enormous quantity of CdSe/ZnS QDs, which significantly amplifies the QDs’ ECL signals. This makes the study beneficial. Using QDs, which had their surfaces coupled with certain peptides, blood vessels, and cancer cells were targeted [27]. Because the QD antibody binds to tumor-specific antigens well, an ABC triblock copolymer-encapsulated QD was shown previously in 2004 to be able to scan biomarkers for human prostate cancer in animals [28].

Upon coating CdSe/ZnS QDs with mercaptoacetic acid, they were covalently linked to the transferrin protein, and the spontaneously endocytosed QDs produced by cancerous HeLa cells maintained their strong fluorescence, suggesting that QDs may be employed as intracellular markers [29]. Folic acid (FA), which is folate in its oxidized state, has a strong affinity for binding to folate receptors (K_d_−10^–10^ M) [30]. Since FR is often not present in healthy tissue but is overexpressed in these carcinoma cells, the overexpression of the membrane-associated folic acid receptor (FR) renders FA a potential diagnostic for a range of carcinomas, including ovarian, prostate, and breast cancers [31].

BSA (bovine serum albumin) was identified as a bridge molecule to produce FA-BSA-CdTe/ZnS QD, a combination for cancer diagnostics. Tumors with abnormal expression of FR were diagnosed. This study concluded that FA-BSA-QDs had higher selectivity than only BSA-coated QDs. Most epithelial cells contain a protein called glycoprotein mucin 1 (MUC1), which is a useful biomarker for the early diagnosis of cancer. Designing Cheng et al. a primer, a reporter with a QD label, along with the MUC1 aptamer stem to form a three-component DNA system [32].

Where the glycoprotein mucin 1 (MUC1) peptide is specifically detected by the fluorescent QDs. When the glycoprotein mucin 1 (MUC1) peptide was present, the level of fluorescence was not as high as when it was not. As a result, MUC1 might be found in nanomolar concentration. The type of cancer that strikes youngsters in equatorial Africa most commonly [33] is Burkitt’s lymphoma, a malignancy of the red blood cells. A human Burkitt’s lymphoma cell is called a Ramos cell. Using a mixture of several malignant cells, the development of an assay for the comprehensive qualitative and quantitative characterization of Ramos cells using CdTe QDs [34] was conducted. Since the surface of the Ramos cells was loaded with a noticeable amount of CdTe QDs tags, the formation of a DNA-CdTe QDs encasement occurred, resulting in strong fluorescence.

### Mechanisms of GQDs in cancer diagnosis and therapy

Graphene quantum dots (GQDs) are unique physicochemical and optical properties that make them extremely suitable for cancer diagnosis. Their tunable fluorescence, substantial stability, and high biocompatibility allow GQDs to be ideal probes for fluorescence imaging of cancerous tissues. Once excited upon irradiation, GQDs directly respond with stable and bright fluorescence signals that can facilitate the real-time visualization of tumor cells with high sensitivity and specificity. In addition, surface functionalization of GQDs with biomolecules (e.g., peptides, antibodies, or nucleic acids) can enable selective recognition of cancer biomarkers to improve accuracy for early diagnosis. The targeted imaging capability allows malignant tissues to be distinguishable from their healthy counterpart, and it becomes a useful mechanism for surgical resection and therapy monitoring after procedures. Furthermore, GQDs’ strong electron transfer capability also amplifies GQDs’ use in multimodal imaging platforms (e.g., photoacoustic and magnetic resonance imaging), expanding the imaging role in the diagnostics of cancer [35].

There is a significant role of GQDs (graphene quantum dots) in targeted cancer therapy beyond imaging, particularly with respect to drug delivery. Their small size and high surface-to-volume ratio facilitate efficient loading of chemotherapeutic drugs and therapeutic biomolecules. The functional groups on the surface of GQDs allow for conjugation with targeting ligands that drive a site-specific delivery approach. This targeted delivery achieves a degree of selective and specific therapeutic delivery to tumor tissues, in relation to a reduction of off-target effects. After entering a cell, GQDs allow for stimulated drug payload release, utilizing specific intracellular stimuli (e.g., pH or redox conditions) that allow for controlled and sustained therapeutic delivery. Besides the drug delivery components, GQDs can also provide theranostic capabilities through their inherent fluorescence characteristics, where administration can facilitate tracking of drug delivery and therapeutic outcomes at the same time. Nonetheless, GQDs also produce intrinsic anticancer effects through reactive oxygen species under light irradiation, establishing the capacity to conduct photodynamic therapy [36]. Overall, GQDs can provide a mechanism to span diagnostics and therapeutics, indicating their ability to serve as a multifunctional platform for precision oncology.

### Advantages of GQDs over conventional methods

Graphene quantum dots (GQDs) offer several distinct advantages compared to traditional diagnostic and therapeutic techniques to treat cancer. GQDs exhibit photostability, which prevents the risk of photobleaching associated with using organic dyes, allowing for continuous imaging without compromising the intensity of the signal for the diagnostic imaging. GQDs have excellent biocompatibility and therefore a lesser likelihood of undesired biological effects related to working with living systems. GQDs have good tunable fluorescence, allowing emission across a wide spectral range simply by changing the size, surface chemistry, or the wavelength of excitation. This ability makes GQDs unique and versatile for multimodal imaging. GQDs contain a large surface area, with multiple functional groups, which serve to chemically modify them to produce targeted delivery and multifunctional therapeutic modalities. All these characteristics of GQDs present them as unique and effective tools and may one day serve as a unique method to perform both diagnostic imaging and therapeutics that advance nanomedicine beyond conventional techniques [37].

### Current challenges of GQDs

The potential of GQDs in human applications is substantial. However, they have outstanding challenges that preclude their application in the human experience. Significant concerns regarding toxicity, especially when synthesizing with heavy metal-based precursors or when cadmium or other ions could leach into biological systems, are a serious concern. A simultaneous challenge concerns synthesis that can lead to scalable and reproducible methods. Current methods suffer from yielding crystalline particles of varying sizes with inconsistent surface properties, limiting their reliability in the clinic. Additionally, translating the use of GQDs into the clinic is still limited or restricted by regulatory issues, particularly as there are reasonable concerns about long-term safety in humans [38]. The above issues highlight the lack of toxicological studies and the lack of standardization in manufacturing protocols to ensure consistency and safety in human applications for biomedicine.

### Prospective views

Repurposing QD technology for clinical diagnostics is our aim. In biological and biomedical research, QDs have shown themselves to be effective fluorescent bio-probes. Because of these qualities, they may be used for in vivo and in vitro research, molecular and cellular imaging, which has led to notable improvements in image-guided administration and cancer detection and treatment of chemotherapy drugs. Photosensitizers are made of QDs in PDT and have taken the place of the tracers in SLNB. Despite being hydrophobic, QD technology is nonetheless not widely used. To properly employ QD technology for diagnostic and clinical applications for targeting, imaging, and drug administration, several challenges must be resolved, including toxicity. The population of cancerous cells can still be measured using these more sensitive, qualitative, and quantitative methods. Targeting metastasis is another option, and localized signal strength can be increased, as well as the monitoring of medication distribution in living tissues. Great precision and accuracy in the early and precise identification of tumor biomarkers, and therefore the process of creation of anti-cancer medications in the future to ultimately eradicate cancer, can be facilitated by QD technology. It could turn out to be a straightforward, quick, and effective platform.

### Biomarkers for carcinoma

Biological compounds known as tumor markers are found in bodily fluids such as blood or tissues and serve as indicators of a disease or condition, according to the National Cancer Institute; few are listed in the Table 1.

**Table 1:** Frequently used biomarkers to identify tumors

Cancer type	Biomarker’s
Invasive duct carcinoma	BRCAI, BRCA2, CA15-3, CA 125, CA 27.29, MUC1, NY-BR-I, ING-I, HER2/NEU, ER/PR
Colorectal cancer	CEA, EGF, p53
Squamous cell carcinoma	SCC
Hepatocellular carcinoma	AFP, CEA
Bronchogenic carcinoma	CEA, CA19-9, SCC, NSE, NY-ESO-I, CYFRA21-1
Cutaneous melanoma	Tyrosinase, NY-ESO-I
Ovarian carcinoma	CA125, HCG, p53, CEA, CA 549, CASA, CA 19-9, CA 15-3, MCA, MOV-I, TAG72
Adenocarcinomas	PSA, PAP

Tumor markers can represent a broad class of biological compounds. These include endogenous lipids, proteins, sugars, nucleic acids, or additional cytogenetic parameters as found in tumor tissues, whose amounts might vary, as well as blood or other body fluids [39, 40]. Any abnormalities or modifications reflect the condition of the tumor and its evolution over time and its response to different therapies. These tumor-associated antigens are valuable tools to diagnose cancer and as indicators of the body’s reaction to a certain disease or therapy [41, 42]. Developing a connection between biomarkers of tumors and clinically proven medicines for early, non-invasive tumors. The most challenging task is identifying the cancer prognosis. Effective tumor markers are actively sought because they can lower cancer-related death rates by facilitating early tumor detection. Many putative tumor indicators have emerged in the last 10 years thanks to advances in nanotechnology, carcinogenesis, and tumor growth.

### Toxicity

Because of their hefty metal content composition, the binary QDs’ possible toxicity is a reason for worry. Cadmium ions may have leaked, which would explain the toxicity. In toxicological studies employing animal models that are relevant to clinical settings, the short- and long-term safety of QDs must be proven, even though such QDs should not be acutely harmful if their polymer covering is durable enough to control the release of cadmium. In normal media conditions, research using cell lines has shown that QDs do not affect cell development. Similarly, short-term QD delivery into animals, such as mice or pigs, seems to have no impact on the hormones or behavior of the animals [43].

However, cadmium does not enter quarantine and stays in the body despite all these precautions, which might raise serious regulatory issues if QDs go through clinical trials. Their use in SLN mapping is an exception; following surgery, QDs and the lymphatic tissue will be removed. Important concerns include how quickly QDs within the tumor can be extracted regardless of whether QDs are expelled or stay resident in the body, and if so, in which tissue(s) and organ(s) need to be answered to clear the path for clinical use. To determine the long-term safety profile of QDs, thorough toxicological studies in suitable animal models must be conducted if QDs are kept in the body.

### Focused on tumor (micro) metastases

Targeting solid primary tumors that are quite big (at least 2 mm) and have well-developed vasculature is best achieved using the passive-targeting strategy that utilizes the EPR effect. An excessively small primary tumor size to have an EPR impact, and that is in the very early stages of development, does not require a considerable blood supply [44]. Consequently, active targeting of QDs or tumor-specific targeting is crucial in these situations. Reduce nonspecific QD absorption by the RES, frequently by appropriately modifying the surface on which conjugated targeting ligands are affixed, to optimize active targeting. The ligands themselves are either big proteins like monoclonal antibodies or tiny compounds like folate or peptides. For a variety of reasons, it is more challenging to illustrate the precise tumor targeting of QDs in vivo than it is on tumor cells grown in vitro.

Limitations for QDs, such as vascular endothelium, which stops extravasation, are created by the intricate anatomical framework and physiology of tissues and organ systems. The degradation of protein-based ligands can lead to the loss of their ability to target. Common conjugation chemistries often limit the control over the attachment sites and molecular orientation of complicated macromolecules, such as antibodies, to QDs, which can result in a partial or whole loss of cell-binding ability. Finally, a very small percentage of cell-targeting ligands are “specific” to tumors; this implies that they merely attach to cancer cells and almost rarely to other cells or to off-target cells at all. To reduce the quantity of false-positive results from QD cancer imaging, it is critical to recognize these optimal ligand-receptor pairings, which necessitate a greater comprehension of cancer biology.

### Improved photodynamic treatment (PDT) with GQD-based nanocomposites

**Fig. 4:**
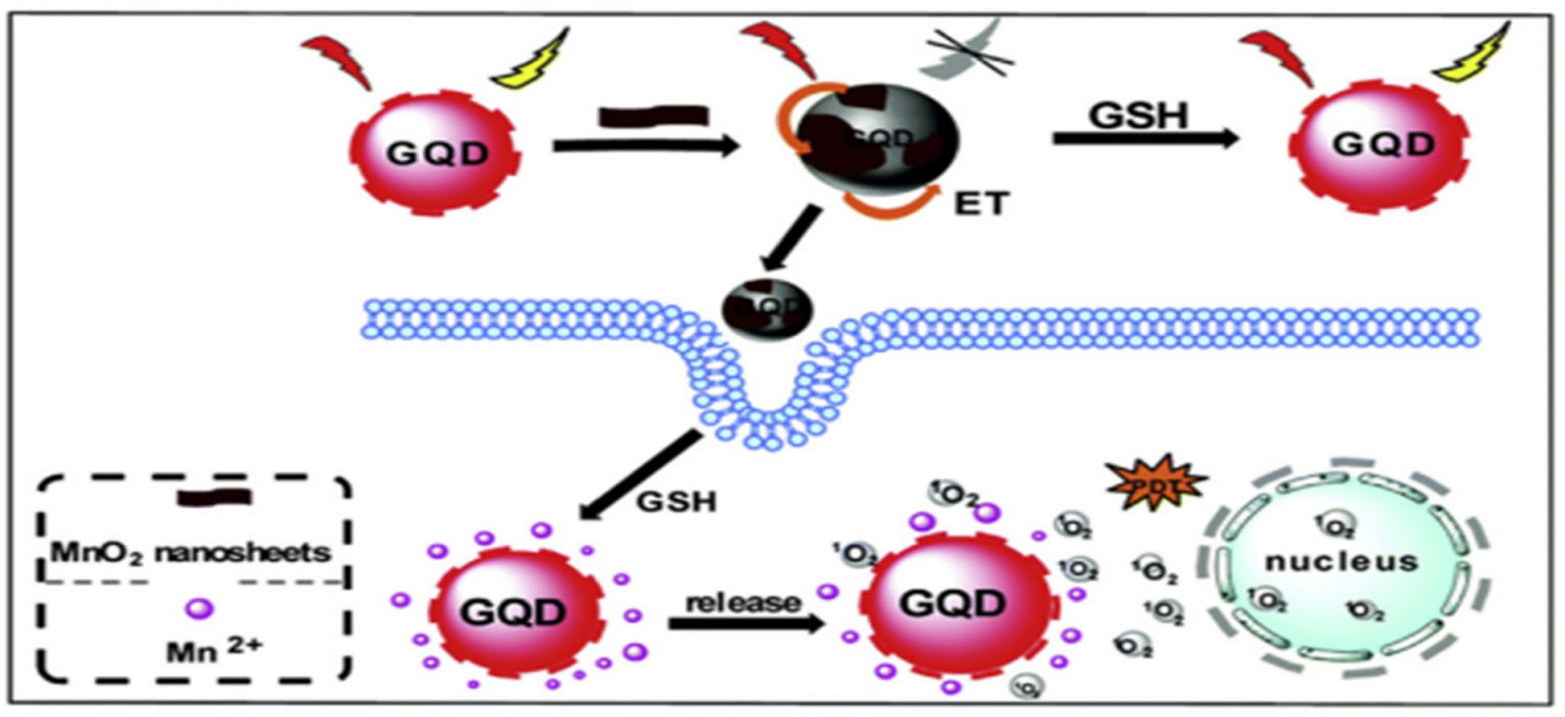
Schematic illustration of GQD@Mno2 with GSH-regulated photoactivity for PDT

PSs and photoexcitation are essential for increasing PDT efficacy since the primary mechanism of PDT includes the production of cytotoxic intracellular ROS by excited PSs in response to photoexcitation. Because GQDs are easily surface functionalized, it is easy to modify them with other biocompatible compounds to get improved PDT. Furthermore, surface functional groups and modifications in structure may have substantial impacts on small-sized GQDs. To acquire the finest PDT agents, it is essential to decorate GQDs with certain attributes. Here, we provide an outline of the GQD-focused nanocomposites that are being developed for improved photodynamic therapy (PDT) illustrated in Fig. 4. These nanocomposites use modified GQD-based nanomaterials as photodynamic therapy (PDT) agents and two-photon excitation (TPE) as an energy source.

GQD-based nanocomposites produced by two-photon excitation for PDT.

In the realm of biological medical imaging and detection, TPE has garnered significant interest [45]. TPE has several benefits over single-photon excitation, including low photodamage in biosystems, great penetration, and precise spatial resolution [46]. Moreover, TPE has been employed to boost PDT therapy effectiveness. The TPE feature of GQD-induced nanomaterials is driving efforts to produce enhanced PDT.

GQD@MnO2, a unique multifunctional two-photo nanoprobe based on GQDs, was created by Meng and colleagues [47], to improve PDT and imaging glutathione (GSH) in biosystems. Being the most common intracellular thiol and a widespread tripeptide, GSH has been demonstrated to consume ^1^O2, significantly reducing PDT's efficacy. Moreover, this effect has been shown in vitro. The efficacy of GQDs on PDT was greatly increased since the GQD@MnO2 nanoprobe showed strong sensitivity and selectivity towards GSH, and intracellular GSH may decrease MnO2 nano-sheets, resulting in a reduced GSH level [48].

Researchers discovered that GQD@MnO2 had a substantially higher PDT effectiveness than GQDs after exposing HeLa cells to 560 nm irradiated for 30 min in an experiment performed in vitro. N-(nitrogen)-doped GQDs have furthermore been developed for enhanced PDT and show strong two-photon properties.

## Conclusion

In addition to having optoelectronic qualities that make them an intriguing advanced material for several applications, graphene quantum dots may be widely accessible. Part of the goal of creating better, more efficient cancer-imaging probes has already been made possible by the quick development of QD technology. Since QDs and biomolecules have successfully conjugated, tumors may now be actively targeted. The issues of optimizing specificity, reducing toxicity, and improving the sensitivity of QDs must be taken care of before clinical applications move forward, despite their potential and current effectiveness in cancer imaging.

## Summary

- Semiconductor nanocrystals known as “quantum dots” glow when excited. They exhibit exceptional optical qualities, including tunable emission spectra, resistance to photobleaching, and great brightness.
- Regarding toxicity and nonspecific reticuloendothelial system absorption, quantum dots have sparked concerns. They also confront difficulties in achieving more precise targeting of metastatic tumors in vivo.
- Advancements in quantum dots might lead to their use in metastatic detection and localization, targeted therapy delivery tracking, quantitative molecular target assessment, and real-time noninvasive therapeutic effectiveness monitoring.

## Data Availability

No datasets were generated or analysed during the current study.

## Abbreviations

AFP: Alfa-feto protein
BSA: Bovine serum albumin
CA: Anhydrous citric acid
C-Dots: Carbon dots
Cd: Cadmium
CdSe: Cadmium selenide
CdS: Cadmium sulfide
CdTe: Cadmium telluride
CKK-8: Cell counting kit -8
CQD: Carbon quantum dots
FA: Folic acid
FA-BSA: Folic acid–bovine serum albumin
FR: Fluorescence resonance
Gd-Lipid: Gadolinium-lipid
GSH: Glutathione
GQD’s: Graphene quantum dots
MUC 1: Mucin 1
MWCNTS: Multi-walled carbon nanotubes
NP’s: Nanoparticles
PDT: Photodynamic therapy
PSA: Prostate-specific antigen
PL Emission: Photoluminescence emission
QD’s: Quantum dots
ECL: Electrochemiluminescence
ROS: Reactive oxygen species
SCC: Self-consistent charge
SLNB: Sentinel lymph node biopsy
ZnS: Zinc sulfide
